# Study of microbiocenosis of canine dental biofilms

**DOI:** 10.1038/s41598-021-99342-5

**Published:** 2021-10-05

**Authors:** Jana Kačírová, Aladár Maďari, Rastislav Mucha, Lívia K. Fecskeová, Izabela Mujakic, Michal Koblížek, Radomíra Nemcová, Marián Maďar

**Affiliations:** 1 Department of Microbiology and Immunology, University of Veterinary Medicine and Pharmacy in Košice, Komenského 73, 041 81 Košice, Slovak Republic; 2 University Veterinary Hospital, University of Veterinary Medicine and Pharmacy in Košice, Komenského 73, 041 81 Košice, Slovak Republic; 3 Institute of Neurobiology, Biomedical Research Center of the Slovak Academy of Sciences, Šoltésovej 4, 040 01 Košice, Slovak Republic; 4 Centre Algatech, Institute of Microbiology of the Czech Academy of Sciences, Novohradská 237, 37901 Třeboň, Czech Republic

**Keywords:** Microbiology, Molecular biology

## Abstract

Dental biofilm is a complex microbial community influenced by many exogenous and endogenous factors. Despite long-term studies, its bacterial composition is still not clearly understood. While most of the research on dental biofilms was conducted in humans, much less information is available from companion animals. In this study, we analyzed the composition of canine dental biofilms using both standard cultivation on solid media and amplicon sequencing, and compared the two approaches. The 16S rRNA gene sequences were used to define the bacterial community of canine dental biofilm with both, culture-dependent and culture-independent methods. After DNA extraction from each sample, the V3–V4 region of the 16S rRNA gene was amplified and sequenced via Illumina MiSeq platform. Isolated bacteria were identified using universal primers and Sanger sequencing. Representatives of 18 bacterial genera belonging to 5 phyla were isolated from solid media. Amplicon sequencing largely expanded this information identifying in total 284 operational taxonomic units belonging to 10 bacterial phyla. Amplicon sequencing revealed much higher diversity of bacteria in the canine dental biofilms, when compared to standard cultivation approach. In contrast, cultured representatives of several bacterial families were not identified by amplicon sequencing.

## Introduction

The oral microbiome in humans has been studied since times of Antonie van Leeuwenhoek. Thanks to easy sampling, it has become one of the most studied microbiomes so far^1–5^. Currently, characteristic bacterial populations of the oral microbiome are known to be present in various individuals. However, the composition of the microbiome can vary greatly from person to person depending on health state, lifestyle, or due to many other factors^6^. Humans influence the natural development of the microbiota of the oral cavity not only by using various oral hygiene products such as toothpastes or mouthwash^7–9^, but also by consuming an unsuitable diet with a high proportion of sugars and carbonated drinks^10^.

Despite the high degree of knowledge in this field of microbiology and dentistry in humans, it is still difficult to define the exact composition of the oral microbiome^11^ and, even more difficult to define it in companion animals. The microbiome of the oral cavity in dogs is influenced mainly by their owners, namely home dental care, but also by the choice of diet^12–14^. There are many owners who neglect oral hygiene in their dogs. Maintaining oral homeostasis is important, especially when it comes to the development of various diseases^5^. The most common manifestation of the imbalance in the proportion of physiologically beneficial and pathological microbiota is the development of diseases such as dental caries^15^, periodontal diseases^16^ and other related diseases even outside the oral cavity^17^.

Previously, the study of the oral microbiome was limited to culture-dependent methods. Sophisticated technologies are currently available to determine the composition of a microbiome in humans or companion animals. The composition of bacterial populations can be defined, even in difficult-to-culture or non-culturable microbiota, with the help of 16S ribosomal RNA gene amplicon sequencing^18^. In this study, we focused on the identification of the oral microbiome of five small breed dogs using both methods.

## Methods

### Animals and samples collection

Samples of dental biofilm were obtained from five dogs of small breeds (Table 1) at the Clinic of Small Animals, University of Veterinary Medicine and Pharmacy in Kosice. Informed consent was obtained from the owner of the dogs for the study. The study is approved by the State Veterinary and Food Administration of the Slovak Republic and by Ethics Commission of the University of Veterinary Medicine and Pharmacy (Kosice, Slovakia). The animals were handled in a humane manner in accordance with the guidelines established by the relevant commission. All applicable international, national and institutional guidelines for the care and use of animals were followed. Before dental biofilm collection, the stage of periodontal disease was assessed by Bauer et al.^19^. Dental biofilm from the buccal surfaces of maxillary canines and maxillary premolars was collected using sterile syringe needle (1.20 × 40 mm, KD-FINE, Berlin, Germany) according to Schaeken et al*.*^20^ with some modifications. Biofilm from the syringe needle was transferred to a DNA LoBind Eppendorf tube (Eppendorf, Hamburg, Germany) containing 300 µl of sterile phosphate-buffered saline. The Eppendorf tubes with samples were vortexed at maximum speed for 20 s and shaken at 400 RPM for 20 min for content homogenization.

**Table 1:** Studied animals. General information on the breed, age and sex of the sampling dogs (D1–D5).

Dog	Breed	Age (years)	Sex
D1	Jack Russell Terrier	13	Male
D2	Maltese	7	Female
D3	Yorkshire Terrier	5	Female
D4	Chihuahua cross	9	Male
D5	Maltese	2	Female

### Cultivable bacterial microbiota of dental biofilms

#### Microbiological cultivation

Aliquots of homogenized samples were decimally serially diluted in phosphate-buffered saline and subsequently volume of 25 µl was inoculated to Trypticase soy agar (TSA; pH 7.2 ± 0.1, Carl Roth GmbH and Co., Karlsruhe, Germany) containing 5% ram’s blood and Mitis Salivarius agar (MSA; pH 7.0 ± 0.2, Sigma Aldrich, Steinheim, Germany) with 1% potassium tellurite solution (Sigma Aldrich, Steinheim, Germany) according to Pieri et al*.*^21^. Samples were cultured under aerobic and anaerobic (BBL GasPak Plus, Becton, Dickinson and Co., Maryland, USA) conditions at 37 °C. After 2 days of aerobic and after 3 days of anaerobic cultivation, individual solitary colonies with different morphological characteristics were selected and subcultured to obtain pure bacterial cultures. After 7 days of anaerobic cultivation, plates were again examined for detection of black-pigmented colonies of *Porphyromonas*. The pure bacterial colonies were transferred to Eppendorf tubes containing Brain Heart Infusion broth (BHI broth; pH 7.4 ± 0.2, HiMedia, Mumbai, India). Subsequently, glycerol (20% v/v) was equally (1:1) added and the isolates were stored at − 80 °C.

#### DNA isolation and amplification

DNA from pure bacterial colonies was isolated by DNAzol direct (Molecular Research Center, Inc., Cincinnati, USA) following the manufacturer's instructions. The 16S rRNA genes from the isolates were amplified by PCR using the universal primers as originally presented by Lane^22^: 27F (5-AGAGTTTGATCMTGGCTCAG-3) and 1492R (5-CGGYTACCTTGTTACGACTT-3) using OneTaq 2X Master Mix with Standard Buffer (New England Biolabs, Foster City, USA). The PCR cycling conditions comprised an initial denaturation phase of 5 min at 94 °C, followed by 30 cycles of denaturation at 94 °C for 1 min, annealing at 55 °C for 1 min and primer extension at 72 °C for 3 min and finally a primer extension step at 72 °C for 10 min. The PCR was conducted in a thermal cycler (TProfesional Basic, Biometra GmbH, Göttingen, Germany). PCR products were visualized with GelRed (Biotium, Inc., Hayward, USA) in 3% agarose gel electrophoresis under ultraviolet light.

#### Sequencing and data analysis

The amplification products were sent for Sanger sequencing using primer 1492R (Microsynth Austria GmbH, Wien, Austria). The obtained chromatograms of sequences were analyzed using Geneious 8.0.5 (Biomatters, Auckland, New Zealand). All isolates were initially identified performing database searches, comparing 16S rRNA sequences obtained from a single reading with sequences available in the GenBank using the Basic Local Alignment Search Tools, nucleotide (BLASTn) (http://www.ncbi.nlm.nih.gov/BLAST/) from the National Center for Biotechnology Information (NCBI). After identification of all isolates, the sequences of the same bacterial species isolated from one dental biofilm were compared and the best quality sequence was selected. The selected sequences were aligned using MUSCLE in Geneious 8.0.5 and Neighbor-Joining tree was constructed from sequences with a length of 1,050 bp using the Geneious 8.0.5 program as well. The bootstrap analysis was chosen for resampling with a replicate number of 100. The selected nucleotide sequences were deposited in GenBank with accession numbers from MT492050 to MT492058, MT506944 and from MT510351 to MT510395.

### Microbiome of the dental biofilms by amplicon sequencing of the 16S rRNA gene

#### Bacterial genomic DNA extraction

Bacterial genomic DNA was extracted from samples by boiling. The samples were centrifuged at 13,600 g for 10 min. The supernatant was discarded and the pellet was resuspended in 500 µl of RNAse free water (PanReac AppliChem, Darmstadt, Germany) and centrifuged at 13,600 g for 10 min. The supernatant was discarded. The pellet was resuspended in 60 µl RNAse free water and heated to 95 °C for 7 min, cooled on ice and centrifuged at 13,600 g for 1 min. The supernatant was transferred to Eppendorf tubes and concentration of DNA was measured (NanoDrop 1000, Thermo Fisher Scientific, Waltham, USA). Samples were diluted to concentration 50 ng µl^-1^ of template DNA.

#### Sequencing library preparation

Aliquots of 2 µl of template DNA were used for PCR. The 16S rRNA gene library was prepared using universal primers targeting the V3–V4 region (460 bp)^23^ using Phusion DNA polymerase (Thermo Fisher Scientific, Waltham, USA). The PCR cycling conditions comprised an initial denaturation phase of 3 min at 98 °C, followed by 25 cycles of denaturation at 98 °C for 10 s, annealing at 60 °C for 20 s and primer extension at 72 °C for 20 s and finally a primer extension step at 72 °C for 3 min. PCR amplifications were performed in triplicate, which were pooled and gel purified using the Wizard SV Gel and PCR Clean-Up System kit (Promega, Madison, USA). The sequencing was performed using Illumina MiSeq platform in (2 × 250 bp) reads at the Genomics Core Facility (Universitat Pompeu Fabra, Barcelona, Spain).

#### Bioinformatic processing of sequencing data

Initial processing of the obtained sequences was carried out in SEED2^24^: reads were joined using the fastq-join function with default settings, the primers were cut off and the sequences were filtered for mean sequence quality ≥ 30 and the correct length of the amplicon (approx. 420 bp). A chimera check was performed using UPARSE (built in SEED2)^25^. Further processing was carried out using the Silva NGS online platform (https://www.arb-silva.de/ngs/), with operational taxonomic unit clustering threshold set at 98% similarity and other settings left at default. The raw unpaired sequence reads were submitted to the NCBI database under BioProject identification number PRJNA634889.

## Results

### Periodontal status of the dogs

An oral examination in dogs was revealed redness and swelling of the gums in all sampling dogs. Also, the presence of tartar was observed in all dogs. Base on this fact, all dogs were included in the III stage of periodontal disease.

### Detected cultivable bacteria

**Figure 1:**
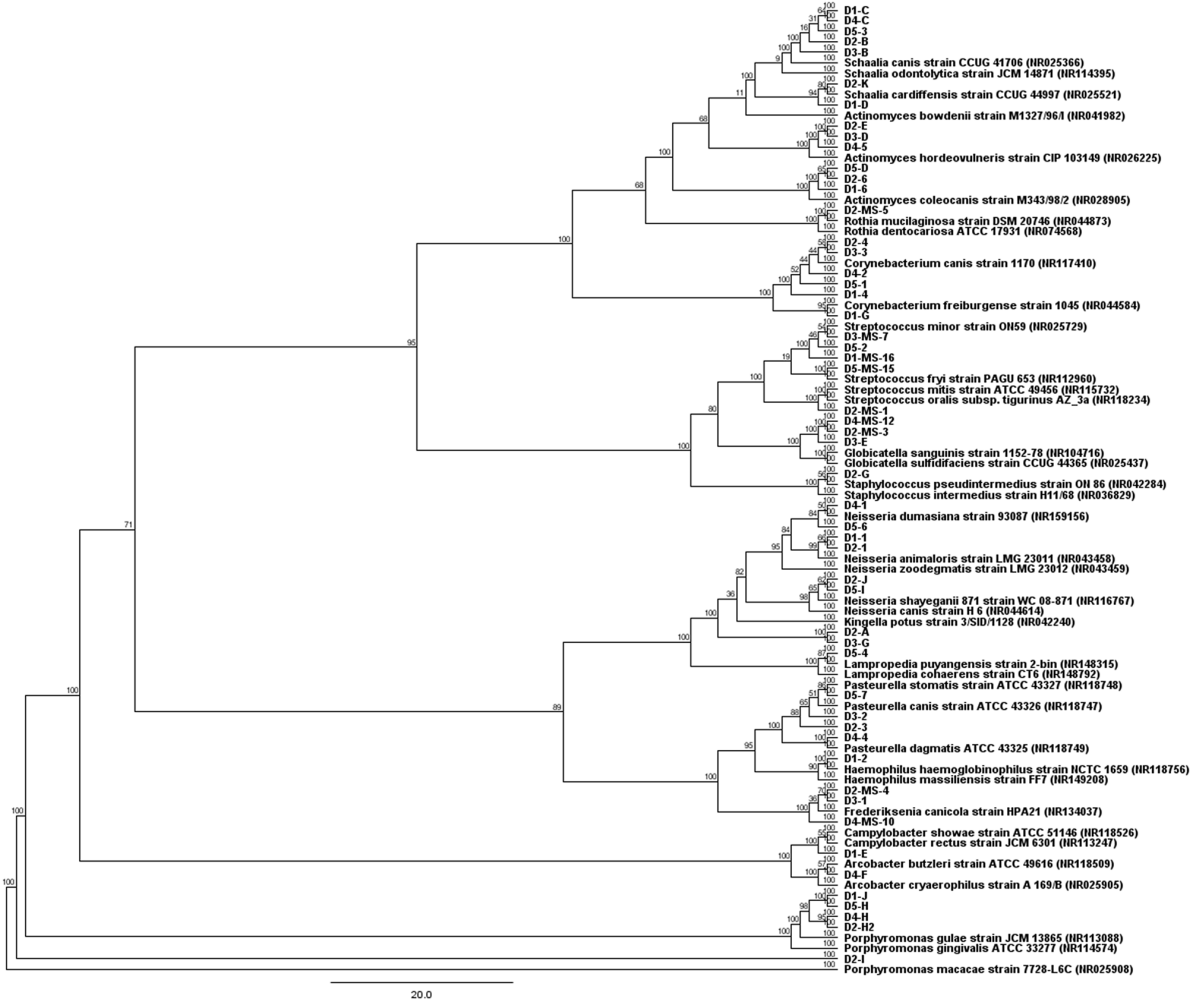
Phylogenetic tree of selected bacterial strains isolated from canine dental biofilms. Phylogenetic tree was made using the neighbor joining method, with 100 bootstrap replicates. Designations D1 to D5 indicate individual dogs, MS indicate Mitis Salivarius agar and numbers indicate individual isolates. The GenBank accession numbers for sequences obtained from the NCBI database are shown in parentheses.

Based on the size, color and growth form of the solitary colonies, 85 strains were selected and sequenced. After sequencing and comparison of individual 16S rRNA genes sequences, 55 unique sequences were selected and subsequently a phylogenetic tree was constructed (Fig. 1).

Through culture-dependent methods, 55 strains were detected belonging to 5 phyla. The highest number of bacterial strains was obtained from D2 (*n* = 16), followed by D5 (*n* = 11), D1 (*n* = 10), D4 (*n* = 10) and the lowest number of bacterial strains from D3 (*n* = 8). The majority of strains belonged to phyla Actinobacteria (38.18%) and Proteobacteria (32.73%). The phyla Firmicutes and Bacteroidetes were represented by 16.36% and 9.09%, respectively. The phylum Epsilonbacteraeota was represented by only 3.64%. Actinobacteria were represented by 5 genera, Proteobacteria were represented by 7 genera, Firmicutes were represented by 3 genera, Epsilonbacteraeota were represented by 2 genera and Bacteroidetes were represented by 1 genus. Overall, strains belonged to 18 genera, namely *Schaalia* (12.73%), *Corynebacterium* (10.91%), *Neisseria* (10.91%), *Porphyromonas* (9.09%), *Streptococcus* (9.09%), *Actinomyces* (7.27%), *Pasteurella* (7.27%), *Frederiksenia* (5.45%), *Gleimia* (5.45%), *Globicatella* (5.45%), *Kingella* (3.64%), *Actinobacillus* (1.82%), *Arcobacter* (1.82%), *Campylobacter* (1.82%), *Haemophilus* (1.82%), *Lampropedia* (1.82%), *Rothia* (1.82%) and *Staphylococcus* (1.82%). Most strains were isolated from Trypticase soy agar containing 5% ram’s blood. In addition to the genus *Streptococcus*, genera *Globicatella*, *Frederiksenia* and *Rothia* were also isolated from Mitis Salivarius agar.

### Overall bacterial composition

172 304 sequences (average sequence number/sample ± standard deviation: 34,460 ± 3012) were divided in 284 operational taxonomic units (OTUs) at 98% clustering threshold. In total, 0.4% of sequences could not be assigned to any taxons. For further calculations, only OTUs representing at least 0.1% of sequences in a given sample in at least one sample, were selected, which resulted in 66 OTUs. OTUs comprise of 10 phyla, 17 classes, 22 orders, 44 families and 66 genera. Alpha diversity analysis identified samples D5 and D2 as the most diverse samples with the highest numbers of OTUs and diversity indices (Supplementary Table S1). Rarefaction curves depict sampling efforts as efficient and comparable in all samples (Supplementary Fig. S1).

**Figure 2:**
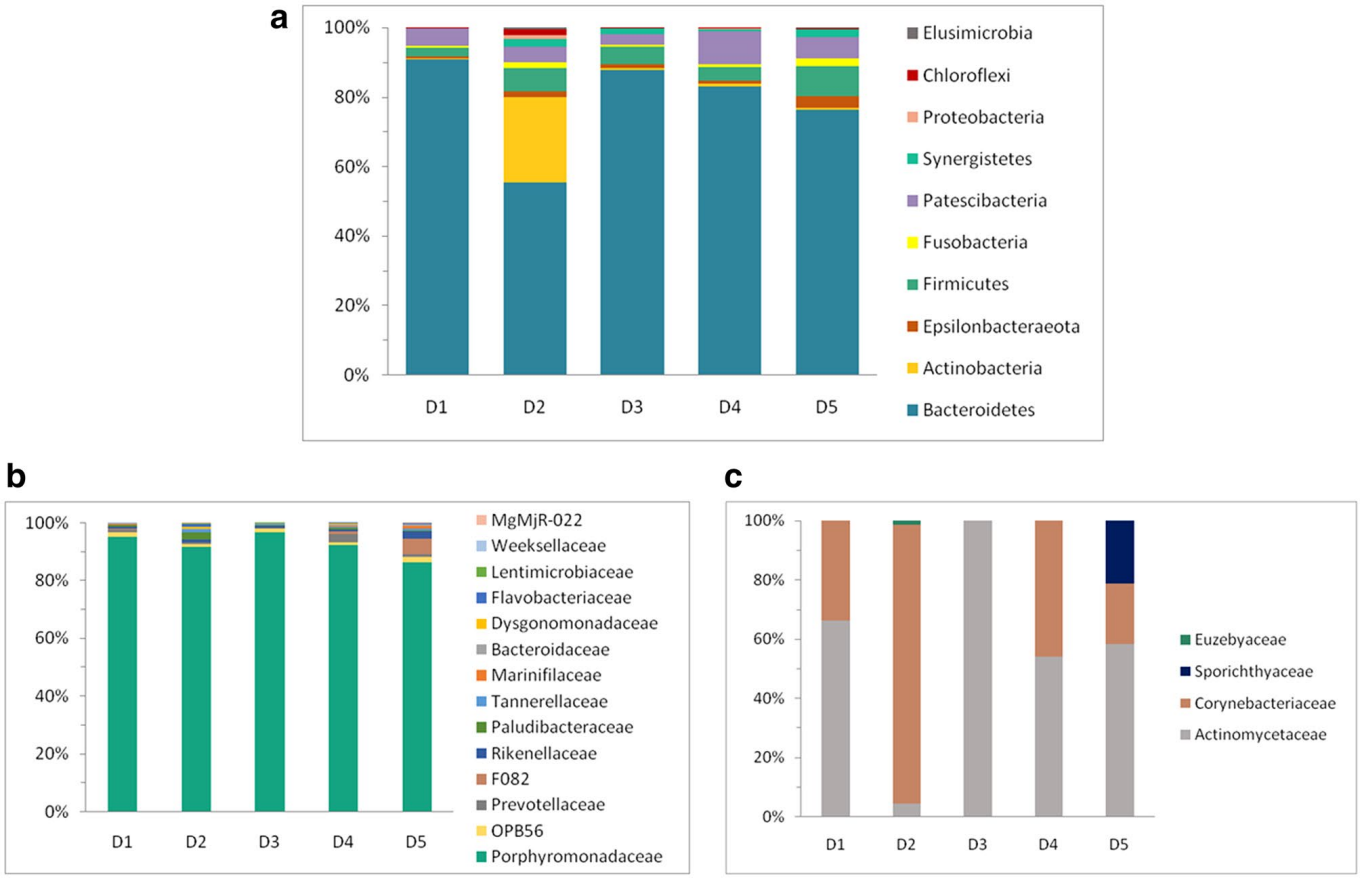
Composition of total bacterial community of canine dental biofilms. (**a**) Composition of total bacterial community at the phylum level, (**b**) families of phylum Bacteroidetes, (**c**) families of phylum Actinobacteria.

Identified phylogroups fell into 10 bacterial phyla as follows based on their relative abundance: Bacteroidetes, Actinobacteria, Firmicutes, Patescibacteria, Epsilonbacteraeota, Fusobacteria, Synergistetes, Chloroflexi, Proteobacteria and Elusimicrobia (Fig. 2a). OTUs assigned to Bacteroidetes (Fig. 2b) dominated the community in all samples, representing between 50 and 90% of the sequences. *Porphyromonas* (periodontal disease-associated genus) was the main genus of the group. In sample D2 Actinobacterial OTUs (Fig. 2c) represented a substantial portion of the community (24%) with *Corynebacterium* as a representative genus, while in all the other samples OTUs assigned to this phylum were only a minor portion. Firmicutes were found in abundances between 2.6 and 8.4% with the most abundant families *Peptostreptococcaceae*, *Family XII*, *Christensenellaceae*, *Lachnospiraceae* and *Ruminococcaceae*. Patescibacteria were the second most abundant phyla in sample D4 with 9.4% of sequences, but in other samples Patescibacteria were substantially less represented (2.9–6%). Proteobacteria were only a minor portion of the community, usually below 0.5%, except for the sample D2, where they were around 1%. The most abundant OTUs identified in the samples are shown in Table 2.

**Table 2:** The most abundant operational taxonomic units detected in canine dental biofilms.

Assigned Taxonomy (Phylum; Class; Order; Family; Genus)	Proportion of total sequence reads (%)				
	D1	D2	D3	D4	D5
Bacteroidetes; Bacteroidia; Bacteroidales; Porphyromonadaceae; *Porphyromonas*	84.69	49.70	82.95	73.28	63.52
Actinobacteria; Actinobacteria; Corynebacteriales; Corynebacteriaceae; *Corynebacterium*	0.13	22.99	0.00	0.28	0.11
Patescibacteria; Gracilibacteria; Absconditabacteriales (SR1)	3.18	1.81	2.16	4.94	4.25
Firmicutes; Clostridia; Clostridiales; Family XII; *Fusibacter*	0.52	0.94	1.73	1.00	3.20
Synergistetes; Synergistia; Synergistales; Synergistaceae; *Fretibacterium*	0.18	2.27	1.76	0.61	2.09
Epsilonbacteraeota; Campylobacteria; Campylobacterales; Campylobacteraceae; *Campylobacter*	0.47	1.54	0.96	0.86	3.03
Fusobacteria; Fusobacteriia; Fusobacteriales; Fusobacteriaceae; *Fusobacterium*	0.37	1.74	0.23	0.95	1.99
Bacteroidetes; Ignavibacteria; OPB56	1.58	0.48	1.11	0.85	1.27
Bacteroidetes; Bacteroidia; Bacteroidales; F082	0.19	0.00	0.05	0.71	3.89
Firmicutes; Clostridia; Clostridiales; Christensenellaceae; Christensenellaceae R-7 group	1.16	1.77	0.43	0.77	0.29
Bacteroidetes; Bacteroidia; Bacteroidales; Rikenellaceae; Rikenellaceae RC9 gut group	0.49	0.60	0.58	0.61	1.74
Firmicutes; Clostridia; Clostridiales; Peptostreptococcaceae; uncultured	0.00	0.81	0.50	0.59	1.90
Bacteroidetes; Bacteroidia; Bacteroidales; Prevotellaceae; *Alloprevotella*	0.00	0.36	0.33	1.83	0.71
Patescibacteria; Parcubacteria; Candidatus Moranbacteria	0.00	0.00	0.00	2.14	0.83
Patescibacteria; Saccharimonadia; Saccharimonadales; Saccharimonadaceae	0.42	0.88	0.00	1.42	0.13
Bacteroidetes; Bacteroidia; Bacteroidales	0.00	0.00	0.00	2.23	0.46
Chloroflexi; Anaerolineae; Anaerolineales; Anaerolineaceae; *Flexilinea*	0.12	1.66	0.25	0.24	0.16
Actinobacteria; Actinobacteria; Actinomycetales; Actinomycetaceae; *Actinomyces*	0.26	1.03	0.30	0.33	0.30
Bacteroidetes; Bacteroidia; Bacteroidales; Paludibacteraceae; F0058	0.35	1.11	0.00	0.19	0.14

Designation D1 to D5 indicate individual dogs. The percent abundance refers to the proportion of 19 OTUs that represent at > 1% of total sequence reads present in at least one sample.

### Comparison of results obtained from standard cultivation on solid media and amplicon sequencing

Using 16S rRNA gene amplicon sequencing 10 bacterial phyla were detected, while only 5 phyla (Actinobacteria, Bacteroidetes, Epsilonbacteraeota, Proteobacteria and Firmicutes) were detected using cultivation on solid media. Phyla Chloroflexi, Elusimicrobia, Fusobacteria, Patescibacteria and Synergistetes were detected only by amplicon sequencing. Representatives of the genus *Porphyromonas*, which dominated in all samples using amplicon sequencing, were detected in 4 samples using microbial cultivation. Non-cultivable bacteria, but also several cultivable bacteria such as representatives of genera *Bergeyella*, *Prevotella*, *Moraxella* or *Wolinella* were not detected using microbial cultivation. On the other hand families Micrococcaceae (genus *Rothia*), Staphylococcaceae (genus *Staphylococcus*), Aerococcaceae (genus *Globicatella*), Streptococcaceae (genus *Streptococcus*), Comamonadaceae (genus *Lampropedia*), Neisseriaceae (genus *Kingella* and *Neisseria*) and Pasteurellaceae (genus *Actinobacillus*, *Frederiksenia*, *Haemophilus* and *Pasteurella*) were detected only by microbial cultivation. The comparison of the detected families in the individual samples by both methods is shown in Table 3.

**Table 3:** Comparison of detection of individual families using microbial cultivation and amplicon sequencing.

Assigned taxonomy family (Phylum)	MC D1	AS D1	MC D2	AS D2	MC D3	AS D3	MC D4	AS D4	MC D5	AS D5
Actinomycetaceae (Actinobacteria)	D	D	D	D	D	D	D	D	D	D
Micrococcaceae (Actinobacteria)	ND	ND	D	ND	ND	ND	ND	ND	ND	ND
Corynebacteriaceae (Actinobacteria)	D	D	D	D	D	ND	D	D	D	D
Sporichthyaceae (Actinobacteria)	ND	ND	ND	ND	ND	ND	ND	ND	ND	D
Euzebyaceae (Actinobacteria)	ND	ND	ND	D	ND	ND	ND	ND	ND	ND
Bacteroidaceae (Bacteroidetes)	ND	D	ND	ND	ND	D	ND	D	ND	D
Dysgonomonadaceae (Bacteroidetes)	ND	ND	ND	D	ND	ND	ND	D	ND	D
F082 (Bacteroidetes)	ND	D	ND	ND	ND	ND	ND	D	ND	D
Marinifilaceae (Bacteroidetes)	ND	D	ND	D	ND	D	ND	D	ND	D
MgMjR-022 (Bacteroidetes)	ND	ND	ND	ND	ND	ND	ND	ND	ND	D
Paludibacteracea (Bacteroidetes)	ND	D	ND	D	ND	D	ND	D	ND	D
Porphyromonadaceae (Bacteroidetes)	D	D	D	D	ND	D	D	D	D	D
Prevotellaceae (Bacteroidetes)	ND	D	ND	D	ND	D	ND	D	ND	D
Rikenellaceae (Bacteroidetes)	ND	D	ND	D	ND	D	ND	D	ND	D
Tannerellaceae (Bacteroidetes)	ND	D	ND	D	ND	D	ND	D	ND	D
Flavobacteriaceae (Bacteroidetes)	ND	ND	ND	D	ND	ND	ND	D	ND	D
Weeksellaceae (Bacteroidetes)	ND	D	ND	D	ND	ND	ND	ND	ND	ND
Lentimicrobiaceae (Bacteroidetes)	ND	D	ND	D	ND	D	ND	ND	ND	ND
Anaerolineaceae (Chloroflexi)	ND	D	ND	D	ND	D	ND	D	ND	D
Endomicrobiaceae (Elusimicrobia)	ND	ND	ND	D	ND	ND	ND	ND	ND	D
Campylobacteraceae (Epsilonbacteraeota)	D	D	ND	D	ND	D	D	D	ND	D
Helicobacteraceae (Epsilonbacteraeota)	ND	ND	ND	ND	ND	ND	ND	ND	ND	D
Staphylococcaceae (Firmicutes)	ND	ND	D	ND	ND	ND	ND	ND	ND	ND
Aerococcaceae (Firmicutes)	ND	ND	D	ND	D	ND	D	ND	ND	ND
Streptococcaceae (Firmicutes)	D	ND	D	ND	D	ND	ND	ND	D	ND
Christensenellaceae (Firmicutes)	ND	D	ND	D	ND	D	ND	D	ND	D
Clostridiales vadinBB60 group (Firmicutes)	ND	ND	ND	ND	ND	D	ND	D	ND	D
Defluviitaleaceae (Firmicutes)	ND	D	ND	D	ND	D	ND	D	ND	D
Family XI (Firmicutes)	ND	D	ND	D	ND	D	ND	D	ND	D
Family XII (Firmicutes)	ND	D	ND	D	ND	D	ND	D	ND	D
Family XIII (Firmicutes)	ND	ND	ND	D	ND	D	ND	D	ND	D
Lachnospiraceae (Firmicutes)	ND	ND	ND	D	ND	ND	ND	ND	ND	D
Peptostreptococcaceae (Firmicutes)	ND	ND	ND	D	ND	D	ND	D	ND	D
Ruminococcaceae (Firmicutes)	ND	ND	ND	D	ND	D	ND	ND	ND	D
Fusobacteriaceae (Fusobacteria)	ND	D	ND	D	ND	D	ND	D	ND	D
Leptotrichiaceae (Fusobacteria)	ND	D	ND	ND	ND	D	ND	ND	ND	D
Saccharimonadaceae (Patescibacteria)	ND	D	ND	D	ND	D	ND	D	ND	D
Comamonadaceae (Proteobacteria)	ND	ND	ND	ND	ND	ND	ND	ND	D	ND
Neisseriaceae (Proteobacteria)	D	ND	D	ND	D	ND	D	ND	D	ND
Desulfobulbaceae (Proteobacteria)	ND	ND	ND	D	ND	ND	ND	ND	ND	ND
Desulfovibrionaceae (Proteobacteria)	ND	ND	ND	ND	ND	ND	ND	ND	ND	D
Burkholderiaceae (Proteobacteria)	ND	D	ND	ND	ND	ND	ND	ND	ND	ND
Pasteurellaceae (Proteobacteria)	D	ND	D	ND	D	ND	D	ND	D	ND
Moraxellaceae (Proteobacteria)	ND	ND	ND	D	ND	ND	ND	ND	ND	ND
Synergistaceae (Synergistetes)	ND	D	ND	D	ND	D	ND	D	ND	D

Designation D1 to D5 indicate individual dogs.

*MC* Microbial cultivation, *AS* Amplicon sequencing, *D* Detected, *ND* Not Detected.

## Discussion

Previous studies of the canine oral microbiome have used either culture-dependent^21,26^ or culture-independent methods^27,28^. The present study analyzed the microbial composition of the canine dental biofilm using both methods. Analysis of the composition of the bacterial community revealed that genera *Actinomyces*, *Campylobacter*, *Corynebacterium*, *Haemophilus*, *Lampropedia*, *Neisseria*, *Pasteurella*, *Porphyromonas*, *Rothia* and *Streptococcus*, detected in the present study, were also detected in canine dental plaque in the study Elliott et al.^26^. Genus *Staphylococcus*, detected in the present study, was not found in dental plaque samples, but only in saliva samples in the study Elliott et al.^26^. In the study Pieri et al.^21^ targeted on a cultivable microbiota of canine dental plaque using MSA, a wider spectrum of bacteria was isolated and identified than in the present study from MSA. A possible reason for the different results is cultivation under different conditions. In the study of Pieri et al*.*^21^ microaerophilic environment was used, while the present study used an aerobic environment.

In addition to the mentioned genera, the genera *Actinobacillus* (formerly *Pasteurella*), *Gleimia* (formerly *Actinomyces*), *Schaalia* (formerly *Actinomyces*), *Arcobacter*, *Frederiksenia*, *Globicatella* and *Kingella* were also detected in the present study. *Frederiksenia* and *Globicatella* were isolated from both TSA containing 5% ram’s blood and MSA, while *Gleimia*, *Schaalia*, *Actinobacillus*, *Arcobacter* and *Kingella* were only isolated from TSA containing 5% ram’s blood. The presence of *Arcobacter* and *Globicatella* in the canine oral microbiome was also confirmed in the study Dewhirst et al.^27^. The species *Frederiksenia canicola* was described for the first time by Korczak et al*.*^29^. It was later proven that *F*. *canicola* exhibits synergistic biofilm growth with *Porphyromonas gulae*^30^. The species *Kingella potus* was first time isolated from a wound infection caused by the bite of a kinkajou^31^. In the present study, this species was isolated from two female dogs from TSA containing 5% ram’s blood.

In the study Ruparell et al*.*^28^, examining the oral microbiome of dogs, OTUs were identified that belonged to nine phyla: Proteobacteria (32.8%), Firmicutes (27.5%), Bacteroidetes (17.5%), Actinobacteria (4.5%), Fusobacteria (2.0%), Synergistetes (1.7%), Spirochaetes (0.7%), Tenericutes (0.5%) and Chlorobi (0.1%) and four candidate phyla: Saccharibacteria (3.8%), Absconditabacteria (1.6%), Gracilibacteria (0.6%) and WS6 (0.5%). Phyla representing more than 1% of the bacterial community in the aforementioned study were also identified in the present study. In other study, Dewhirst et al*.*^27^, aimed to identify bacterial species present in the canine subgingival plaque using culture-independent methods, were detected 14 bacterial phyla, namely Firmicutes, Proteobacteria, Bacteroidetes, Spirochaetes, Synergistetes, Actinobacteria, Fusobacteria, TM7, Tenericutes, GN02, SR1, Chlorobi, Chloroflexi and WPS-2. Our results were compared with results obtained from the studies by Ruparell et al*.*^28^ and Dewhirst et al.^27^. Phyla Bacteroidetes, Actinobacteria, Firmicutes, Fusobacteria, Synergistetes and Proteobacteria were detected by both researchers as well as in the present study. The phylum Elusimicrobia was detected in the present study and also detected in other studies that examined canine plaque^32,33^. In addition to the previously mentioned phyla, the phylum Epsilonbacteraeota, formerly a class of the phylum Proteobacteria^34^, was detected in the present study. Bacterial candidate phyla Gracilibacteria (formerly known as GN02), Parcubacteria (also known as OD1), WS6 (also known as Dojkabacteria) and class Saccharimonadia (initially described as Candidate division TM7) belonging to the new bacterial superphyla Candidate Phyla Radiation (CPR)/Patescibacteria^35^ were also detected in the present study.

Samples of dental biofilm were collected from dogs with clinical signs of early stages of periodontal disease (gingivitis). The main genus in all samples was *Porphyromonas*, commonly associated with periodontal disease in companion animals. Two species of this genus were isolated from the samples, namely *Porphyromonas gulae* and *Porphyromonas macacae*. *P*. *gulae* has similar virulence factors to the human periodontal pathogen *Porphyromonas gingivalis*, which includes the lysyl- and arginyl-specific proteolytic activity. This finding suggests that *P*. *gulae* may play a key role in the development of periodontitis in dogs^36^. Furthermore, the genera *Fusibacter*, *Fretibacterium*, *Campylobacter* and *Fusobacterium* were present in all samples. *Campylobacter rectus* and *Fusobacterium nucleatum* have been associated with periodontal disease in humans for a long time^37–39^. *Fusobacterium canifelinum* and *Campylobacter rectus* belong to predominant bacterial species in canine dental plaque^40,41^. However, their role in periodontal disease in dogs is not fully understood. It has recently been discovered that uncultivable *Fretibacterium* can be involved in periopathogenesis in humans. *Fretibacterium* was significantly higher in periodontitis group than in the healthy group^42^.

The use of 16S rRNA amplicon sequencing technologies has significantly improved our understanding of microbiomes, including the canine oral microbiome. While analysis of diverse microbial communities using amplicon sequencing is more accurate than traditional culture-based methods, experimental bias introduced during critical steps such as DNA extraction may compromise the results obtained^43^. Detection of species depends on obtaining DNA that can be amplified. The lysis techniques used in the present study did not detect difficult to lyse Gram-positive microorganisms, such as species of *Staphylococcus* and *Streptococcus*, using culture-independent methods. On the other hand, representatives of mentioned genera were detected by microbial cultivation.

In conclusion, amplicon sequencing has revealed a much bigger bacterial diversity in the canine dental biofilms compared to microbial cultivation. In addition, amplicon sequencing provides a rough estimate of the relative abundance of individual phylotypes in the studied community, which helps us understand the relative proportion of cultivable bacteria in the oral biofilm. However, obtaining strains by cultivation still remains necessary for their further research. Therefore, we recommend that these two approaches be used in parallel for research into oral biofilms.

## Supplementary Information

Supplementary Figure S1.

Supplementary Table S1.

## References

[1] Syed SA, Loesche WJ (1973). Efficiency of various growth media in recovering oral bacterial flora from human dental plaque. Appl. Microbiol..

[2] Aas JA, Paster BJ, Stokes LN, Olsen I, Dewhirst FE (2005). Defining the normal bacterial flora of the oral cavity. J. Clin. Microbiol..

[3] Dewhirst FE (2010). The human oral microbiome. J. Bacteriol..

[4] Sizova MV (2012). New approaches for isolation of previously uncultivated oral bacteria. Appl. Environ. Microbiol..

[5] Deo PN, Deshmukh R (2019). Oral microbiome: Unveiling the fundamentals. J. Oral Maxillofac. Pathol..

[6] Willis JR, Gabaldón T (2020). The human oral microbiome in health and disease: From sequences to ecosystems. Microorganisms.

[7] Prasanth M (2011). Antimicrobial efficacy of different toothpastes and mouthrinses: An in vitro study. Dent. Res. J. (Isfahan).

[8] Adams SE (2017). A randomised clinical study to determine the effect of a toothpaste containing enzymes and proteins on plaque oral microbiome ecology. Sci. Rep..

[9] Bescos R (2020). Effects of Chlorhexidine mouthwash on the oral microbiome. Sci. Rep..

[10] Mishra MB, Mishra S (2011). Sugar-sweetened beverages: General and oral health hazards in children and adolescents. Int. J. Clin. Pediatr. Dent..

[11] Sharma N, Bhatia S, Sodhi AS, Batra N (2018). Oral microbiome and health. AIMS Microbiol..

[12] Gawor JP (2006). Influence of diet on oral health in cats and dogs. J. Nutr..

[13] Buckley C (2011). The impact of home-prepared diets and home oral hygiene on oral health in cats and dogs. Br. J. Nutr..

[14] Enlund KB (2020). Dental home care in dogs: A questionnaire study among Swedish dog owners, veterinarians and veterinary nurses. BMC Vet. Res..

[15] Hale FA (2009). Dental caries in the dog. Can. Vet. J..

[16] Riggio MP, Lennon A, Taylor DJ, Bennett D (2011). Molecular identification of bacteria associated with canine periodontal disease. Vet. Microbiol..

[17] Stella JL, Bauer AE, Croney CC (2018). A cross-sectional study to estimate prevalence of periodontal disease in a population of dogs (Canis familiaris) in commercial breeding facilities in Indiana and Illinois. PLoS ONE.

[18] Caselli E (2020). Defining the oral microbiome by whole-genome sequencing and resistome analysis: The complexity of the healthy picture. BMC Microbiol..

[19] Bauer AE, Stella J, Lemmons M, Croney CC (2018). Evaluating the validity and reliability of a visual dental scale for detection of periodontal disease (PD) in non-anesthetized dogs (Canis familiaris). PLoS ONE.

[20] Schaeken MJ, Creugers TJ, Van der Hoeven JS (1987). Relationship between dental plaque indices and bacteria in dental plaque and those in saliva. J. Dent. Res..

[21] Pieri FA, Silva VD, Junior AS, Moreira MA (2018). Cultivable microbiota in mitis salivarius agar from dental plaque of dogs. Anim. Vet. Sci..

[22] Lane DJ, Stackebrandt E, Goodfellow M (1991). 16S/23S rRNA sequencing. Nucleic Acid Techniques in Bacterial Systematics.

[23] Klindworth A (2013). Evaluation of general 16S ribosomal RNA gene PCR primers for classical and next-generation sequencing-based diversity studies. Nucleic. Acids. Res..

[24] Větrovský T, Baldrian P, Morais D (2018). SEED 2: A user-friendly platform for amplicon high-throughput sequencing data analyses. Bioinformatics.

[25] Edgar RC (2013). UPARSE: Highly accurate OTU sequences from microbial amplicon reads. Nat. Methods.

[26] Elliott DR, Wilson M, Buckley CM, Spratt DA (2005). Cultivable oral microbiota of domestic dogs. J. Clin. Microbiol..

[27] Dewhirst FE (2012). The canine oral microbiome. PLoS ONE.

[28] Ruparell A (2020). The canine oral microbiome: Variation in bacterial populations across different niches. BMC Microbiol..

[29] Korczak BM, Bisgaard M, Christensen H, Kuhnert P (2014). Frederiksenia canicola gen. nov., sp. Nov. isolated from dogs and human dog-bite wounds. Antonie Van Leeuwenhoek.

[30] Sanguansermsri P, Nobbs AH, Jenkinson HF, Surarit R (2018). Interspecies dynamics among bacteria associated with canine periodontal disease. Mol. Oral. Microbiol..

[31] Lawson PA (2005). Description of Kingella potus sp. Nov., an organism isolated from a wound caused by an animal bite. J. Clin. Microbiol..

[32] Davis IJ (2013). A cross-sectional survey of bacterial species in plaque from client owned dogs with healthy gingiva, gingivitis or mild periodontitis. PLoS ONE.

[33] Wallis C (2015). A longitudinal assessment of changes in bacterial community composition associated with the development of periodontal disease in dogs. Vet. Microbiol..

[34] Waite DW (2017). Comparative genomic analysis of the class epsilonproteobacteria and proposed reclassification to epsilonbacteraeota (phyl. nov.). Front. Microbiol..

[35] Castelle CJ, Banfield JF (2018). Major new microbial groups expand diversity and alter our understanding of the tree of life. Cell.

[36] Lenzo JC (2016). Porphyromonas gulae has virulence and immunological characteristics similar to those of the human periodontal pathogen porphyromonas gingivalis. Infect. Immun..

[37] Socransky SS, Haffajee AD, Cugini MA, Smith C, Kent RL (1998). Microbial complexes in subgingival plaque. J. Clin. Periodontol..

[38] Macuch PJ, Tanner AC (2000). Campylobacter species in health, gingivitis, and periodontitis. J. Dent. Res..

[39] Signat B, Roques C, Poulet P, Duffaut D (2011). Fusobacterium nucleatum in periodontal health and disease. Curr. Issues Mol. Biol..

[40] Dahlén G, Charalampakis G, Abrahamsson I, Bengtsson L, Falsen E (2012). Predominant bacterial species in subgingival plaque in dogs. J. Periodontal. Res..

[41] Yamasaki Y (2012). Distribution of periodontopathic bacterial species in dogs and their owners. Arch. Oral. Biol..

[42] Khemwong T (2019). Fretibacterium sp. human oral taxon 360 is a novel biomarker for periodontitis screening in the Japanese population. PLoS ONE.

[43] Vesty A, Biswas K, Taylor MW, Gear K, Douglas RG (2017). Evaluating the impact of DNA extraction method on the representation of human oral bacterial and fungal communities. PLoS ONE.

