# Supplementary figures and images for: Study of microbiocenosis of canine dental biofilms
Source: Sci Rep. 2021 Oct 5;11:19776. doi: 10.1038/s41598-021-99342-5 (PMC8492755; doi:10.1038/s41598-021-99342-5)

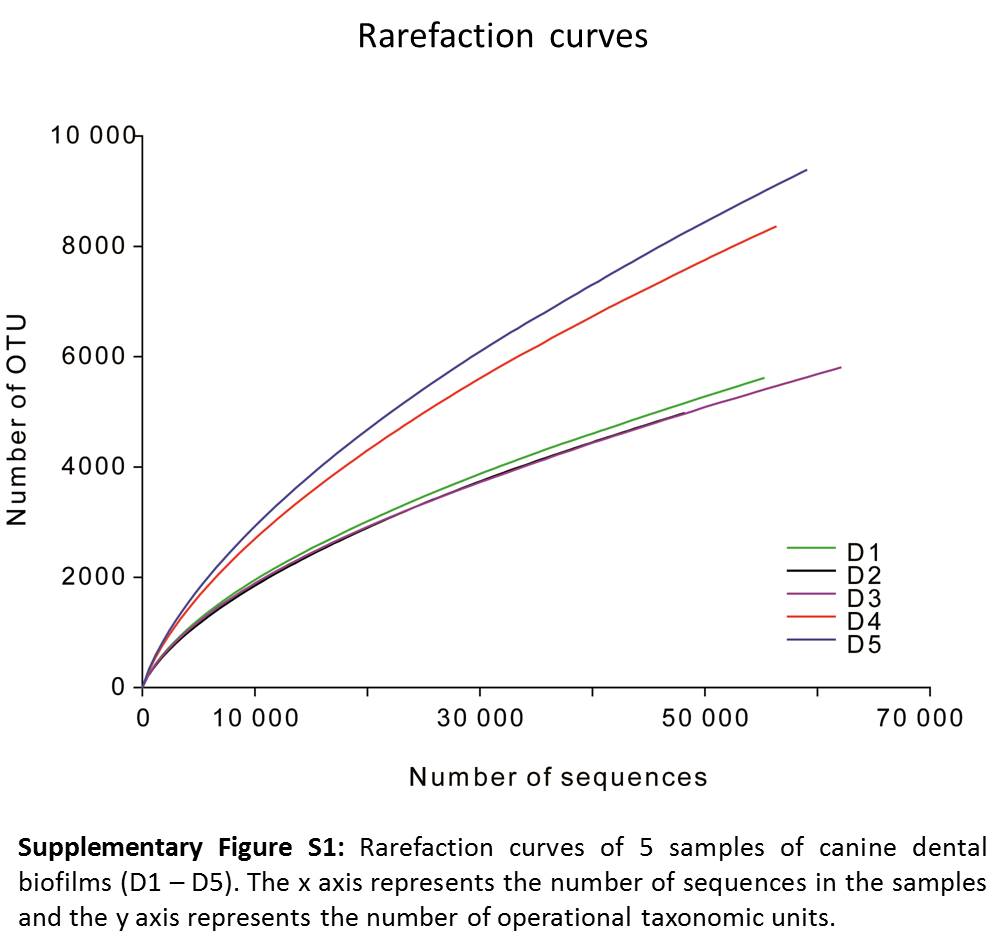

Supplement: Supplementary file 1 — Supplementary Figure S1. [file 41598_2021_99342_MOESM1_ESM.jpg]
